# Sex differences in the clinical outcomes of chronic hepatitis B infection among paediatric patients

**DOI:** 10.1186/s13293-026-00826-8

**Published:** 2026-03-25

**Authors:** Wenxian Ouyang, Zhenzhen Yao, Yingping Gu, Xin Lai, Songxu Peng

**Affiliations:** 1 Liver Disease Center, Hunan Children’s Hospital, Changsha, China; 2 Department of Maternal and Child Health, Xiangya School of Public Health, Central South University, Changsha, 410013 China; 3 Key Laboratory of Hepatosplenic Surgery, Ministry of Education, The First Affiliated Hospital of Harbin Medical University, Harbin, China

**Keywords:** Sex, Treatment response, Difference, Chronic hepatitis B virus infection, Children

## Abstract

**Background:**

The impacts of sex on treatment response in paediatric patients with chronic hepatitis B virus (HBV) infection remains incompletely understood. We aimed to analyse sex differences in antiviral treatment response in children with HBV infection.

**Methods:**

We performed a retrospective cohort study on 236 treatment-naive children with chronic HBV infection at the Liver Disease Centre of Hunan Children’s Hospital. The Cox proportional hazard regression was used to estimated treatment outcome differences between males and females.

**Results:**

Of the 236 subjects, the median age at treatment initiation was 4 (2,7) years, and 155 (65.68%) were male. After the median follow-up (IQR) of 27 (18, 43) months, the crude cumulative incidence of HBsAg loss, HBeAg clearance and HBV DNA undetectability in patients were 48.73%, 61.02% and 89.36%, respectively. Kaplan-Meier curve showed that female patients were more likely to achieving HBsAg loss compared to male patients (58.02% vs. 43.87%, P_log−rank_=0.001). After adjustment for other covariates, female sex was significantly associated with the higher possibility of HBsAg loss (female vs. male: Adjusted HR: 2.03, 95% CI: 1.35–3.06) in children with chronic HBV infection. Furthermore, the landmark analysis revealed that the relationship between sex and HBsAg loss became more pronounced beyond the first year (female vs. male: adjusted HR (95%CI): 2.18(1.29–3.69)).

**Conclusion:**

Children with chronic HBV infection receiving antiviral treatment are frequently observed to achieve functional cure. Sex plays an important role in treatment response among paediatric patients, with female patients exhibiting a higher likelihood of achieving HBsAg loss, particularly after one- year of follow-up.

**Supplementary Information:**

The online version contains supplementary material available at 10.1186/s13293-026-00826-8.

## Highlight

1. This paediatric study reveals a significant sex disparity, demonstrating that girls with chronic HBV are twice as likely as boys to achieve the key treatment goal of HBsAg loss after antiviral therapy.
2. While antiviral treatment frequently led to functional cure outcomes in children with chronic HBV, female patients consistently achieved higher rates of HBsAg clearance, particularly beyond the first year of treatment where their advantage increased significantly.

**Supplementary Information:**

The online version contains supplementary material available at 10.1186/s13293-026-00826-8.

## Abstract (plain language summary)

**What was the research about?**

This study looked at how well antiviral treatments work for children with chronic hepatitis B virus (HBV) infection. It focused on whether being a boy or a girl made a difference in how the children responded to treatment.

**What were the main results?**

Overall, the treatments worked well for many children. However, a key finding was a big difference between boys and girls. After adjusting for other factors that might affect the outcome (like age), the study found that being a girl made a child roughly twice as likely to achieve HBsAg loss compared to being a boy. The advantage for girls increased over time. The difference in cure rates between girls and boys became even more noticeable after the first year of treatment.

**Why is this important?**

This study is one of the first to clearly show that a child’s sex plays an important role in how well they respond to hepatitis B treatment – with girls having a considerable advantage, especially in achieving the goal of a functional cure. This information can help doctors better understand treatment outcomes and perhaps guide future treatment decisions or research for children with chronic hepatitis B.

**Supplementary Information:**

The online version contains supplementary material available at 10.1186/s13293-026-00826-8.

## Introduction

Since the implementation of universal immunization programs, the incidence of hepatitis B virus (HBV) infection in China has decreased significantly [1]. However, chronic HBV infection remains a major public health problem due to the large number of existing infections. The fourth National Serological Survey of China reports that approximately 1.8 million children under the age of 15 have been diagnosed with chronic HBV infection [1]. Remarkably, children have a higher risk of developing chronic HBV infection than adults. The chronicity rates for infections in early childhood and infancy are around 40–50% and over 90% respectively, compared to less than 5% in adults [2]. Chronic HBV infection is strongly associated with the development of HBV-related liver cirrhosis or cancer [3]. Over a lifetime, patients with chronic hepatitis B have a cumulative incidence of hepatocellular carcinoma (HCC) of 9–24% and an annual incidence of cirrhosis of 2–3% [3, 4]. Therefore, it is crucial to prioritise and pay special attention to HBV-infected individuals, especially paediatric patients.

Numerous studies have reported the effectiveness of antiviral therapy for chronic HBV infection in adults. However, the results have been largely disappointing, with many studies reporting unsatisfactory outcomes, particularly in terms of achieving HBsAg loss. Published studies by Hsu and Lau et al. have shown that the incidence of HBsAg loss in treated adults was less than 10% [5, 6]. In contrast, relatively few studies have been conducted on the effectiveness of treatment for chronic HBV infection in the paediatric population. Few studies have suggested that children with chronic HBV infection may have a more favourable response to antiviral treatment [7]. For instance, a real-life cohort study conducted by Hu et al. on 36 children (aged 1–18 years) with chronic HBV infection showed promising results [8]. After treatment with interferon alpha-2b/pegylated interferon alpha-2a, the rates of HBsAg loss, HBeAg seroconversion, and HBV DNA undetectability at 48 weeks of follow-up were 16.7%, 75.0%, and 86.1%, respectively. However, a prospective cohort study conducted by Wirth et al. on 101 chronic HBV-infected children (aged 3 to < 18 years) showed different results [9]. After treatment with PegIFN alpha-2a for 48 weeks, the rates of HBsAg loss, HBeAg clearance, and HBV DNA undetectability at 24 weeks post-treatment were 8.9%, 25.7%, and 16.8%, respectively. These results highlight significant differences in treatment outcomes for chronic HBV infection in paediatric patients between different studies. It suggests that the effectiveness of antiviral treatment for chronic HBV infection in children may be influenced by other factors. Identifying these factors that influence treatment outcomes is therefore crucial for the optimal management of HBV in children.

Previous studies have shown that these factors such as ALT level, HBsAg level, HBeAg status, HBV viral load, and HBV genotype are strongly associated with the efficacy of HBV antiviral therapy [10]. In addition, our previous study also found that the age of initiation of antiviral therapy may also be an influential factor [11]. It is well known that sex plays an important role in the response to treatment for a variety of diseases due to the many biological differences between males and females. However, there are no recent studies on the relationship between sex and antiviral treatment outcomes in chronic HBV infection. Most of the studies only included sex as an adjustment variable in the analyses. Therefore, the effect of sex on antiviral treatment outcomes in chronic HBV-infected children remains unclear. Several studies have reported different responses in the male and female patients with chronic HBV infection after antiviral treatment. For example, in a retrospective cohort study by Mao et al. [12] of 474 chronic HBV-infected children receiving IFN-alpha treatment and in a prospective study by Jonas et al. [13] of 120 treatment-naive HBeAg-positive children with chronic HBV infection receiving entecavir treatment, both studies observed a significant association between sex and antiviral treatment response. In particular, several studies have shown that female patients have better treatment outcomes than males. However, different results were reported by Hom and Liu et al. [14, 15] who found that there was no significant difference in treatment response between males and females. This suggests that more research is needed to fully understand the complexity of this relationship and its potential variation in different populations, especially in chronic HBV-infected children.

Therefore, the aim of this study is to describe the long-term clinical performance of interventions in male and female children with HBV infection. In addition, the assessment of clinical outcomes at the 1-year mark of treatment initiation is commonly used as a mid-term evaluation point for drug efficacy and also serves as a primary endpoint for interferon-based treatment regimens [16], which plays a crucial role in determining subsequent treatment plans. Therefore, the landmark method would be used to further investigate any differences in clinical outcomes before and after the 1-year landmark points. The results of this research would contribute to the development of personalized treatment strategies for children with chronic HBV infection by identifying any sex differences in long-term treatment efficacy.

## Methods

### Study design and participants

A retrospective cohort study was conducted between February 2013 and February 2022 in children diagnosed with HBV infection for more than 6 months at the Liver Disease Centre of Hunan Children’s Hospital. Chronic HBV-infected patients (< 18 years) confirmed HBsAg-positive for > 6 months, who were HBeAg-seropositive and receiving first-time interferon monotherapy, entecavir monotherapy, or combination therapy, were eligible for inclusion. Exclusion criteria were (1) co-infection with other viruses, including hepatitis C virus, hepatitis D virus, Epstein-Barr virus, and cytomegalovirus; (2) diagnosis of other chronic liver-related diseases, including non-alcoholic fatty liver disease, decompensated cirrhosis, and HCC; and (3) less than 12 months of follow-up. Participants received interferon/peginterferon and entecavir monotherapy or combination therapy as clinically indicated, and were monitored for HBV-related markers, including HBsAg, HBeAg, HBV DNA, and ALT levels, every 3–6 months, with more frequent monitoring as necessary to assess treatment response.

### Clinical outcomes and definition

The prespecified follow-up period was every 3–6 months, or more frequently if clinically indicated. In this cohort study, the clinical outcomes included HBsAg loss, defined as a detectable level of less than 0.05 IU/mL, HBeAg clearance, defined as an HBeAg Cut-off Index (COI) of less than 1, and HBV DNA undetectability, defined as an HBV DNA level of less than 100 IU/mL.

### Laboratory tests

Serum HBsAg and HBeAg were determined by electrochemiluminescence (Elecsys HBsAg II, lower detection limit: 0.05 IU/ml; Elecsys HBeAg assay; Roche Diagnostics GmbH, Mannheim, Germany). HBV DNA levels were assessed using a quantitative fluorescence diagnostic HBV kit (lower detection limit: 100 IU/ml; Sanshin Biotech, Changsha, China). HBV genotyping was performed using an HBV genotyping kit (Sanshin Biotech, Changsha, China).

### Statistical analysis

Normally distributed continuous variables were described by the means (standard deviation, SD), whereas skewed variables were described by the medians (interquartile range, IQR). These variables were compared using analysis of variance (ANOVA) or the nonparametric Kruskal-Wallis test. Categorical variables were expressed as numbers (percentages) and compared using the chi-square test or Fisher’s exact test. The Kaplan-Meier method was used to analyse the cumulative incidence of different clinical outcomes, and the log-rank test was used to compare differences between male and female patients. We used Cox proportional hazards regression to estimate the association between sex and clinical outcomes. Additionally, landmark analyses were performed to assess clinical outcomes during different treatment periods. Hazard ratios were calculated for events occurring before and after the 1-year landmark point. Based on an assumed true hazard ratio (HR) of 1.8 for female versus male participants, our power calculation indicated that the current sample size provides the study with 91.6% statistical power. Statistical analyses were performed using R statistical software version 4.1.3 (R Foundation for Statistical Computing, Vienna, Austria), and landmark analyses were performed using the “jskm” package. Two-tailed p-values less than 0.05 were used for all statistical tests.

## Results

### Study population

In this study, a total of 364 treatment-naïve children (aged < 18 years) with chronic HBV infection were included. However, 101 patients were excluded from the analysis because they were followed for less than 12 months. In addition, 27 patients co-infected with other viruses such as hepatitis C, D virus, Epstein-Barr virus or cytomegalovirus, or diagnosed with non-alcoholic fatty liver disease, decompensated cirrhosis, hepatocellular carcinoma (HCC) or other chronic liver diseases were also excluded from the study. Therefore, 236 Paediatric patients with chronic HBV infection were included in the final analysis.

### Baseline characteristics of children with chronic HBV infection by sex

Of the 236 participants, the median age of treatment initiation was 4 (2,7) years old, with 155(65.68%) males. There were 147 (62.29%) patients carrying genotype B and 25 (10.59%) carrying genotype C. 191(80.93%) children received the combination therapy of interferon/peginterferon and entecavir, with a median treatment duration of 23.92(16.84,38.55) months (Table 1).

**Table 1:** Baseline characteristics of children with chronic HBV infection according to sex

Variables	Total	Sex		*P* value
		Male	Female	
No. of participants	236	155	81	
Months of follow-up, median (IQR), mo	27.34(17.61,43.30)	34,16(20.28,47.97)	20.69(16.59,34.94)	<0.001
Age of treatment initiation, median (IQR), y	4.0(2.0,7.0)	4.0(2.0,7.0)	3.0(2.0,6.5)	0.547
Genotype, no.				0.139
B	147(62.29)	89(57.42)	58(71.60)	
C	25(10.59)	19(12.26)	6(7.41)	
Missing, no (%).	64(27.12)	47(30.32)	17(20.99)	
Total bilirubin, median (IQR), umol/L	9.18(6.90,12.19)	9.26(6.90,12.60)	8.79(6.90,11.53)	0.813
Albumin, mean (SD), g/L	42.16(3.42)	42.11(3.51)	42.27(3.25)	0.738
Globulin median (IQR), g/L	25.70(23.20,28.88)	25.70(23.30,29.31)	25.55(23.20,28.48)	0.559
Total bile acide, median (IQR), umol/L	4.43(2.60,7.34)	4.43(2.63,6.99)	4.50(2.49,8.15)	0.924
γ-glutamyltranspeptidase, median (IQR), IU/L	12.00(9.00,22.44)	11.00(8.21,17.67)	14.00(10.00,27.29)	0.003
Platelet count, Mean (SD), ×10^9^/L	281.84(76.63)	281.84(76.63)	273.58(77.29)	0.456
ALT, median (IQR), IU/L	50.80(27.30,93.60)	41.40(22.53,91.00)	54.00(30.20,95.90)	0.040
AST, median (IQR), IU/L	53.80(37.50,90.70)	57.10(38.70,92.57)	47.25(35.86,83.72)	0.085
HBsAb, no.				0.968
Positive	55(23.31)	36(23.23)	19(23.46)	
Negative	181(76.69)	119(76.77)	62(76.54)	
HBsAg, median (IQR), log_10_IU/mL	4.21(2.92,4.59)	4.14(2.79,4.58)	4.29(2.99,4.69)	0.679
HBVDNA, median (IQR), log_10_IU/mL	6.95(5.48,7.60)	7.02(5.67,7.68)	6.80(5.28,7.48)	0.252
Therapeutic regimen				0.083
Interferon/ peginterferon	34(14.41)	27(17.42)	7(8.64)	
Entecavir	11(4.66)	5(3.23)	6(7.41)	
Combination therapy	191(80.93)	123(79.35)	68(83.95)	
Duration of treatment, median (IQR), mo	23.92(16.84,38.55)	27.22(17.31,39.28)	19.69(15.84,31.00)	0.003

ALT, alanine aminotransferase; AST, aspartate aminotransferase; HBsAg, Hepatitis B surface antigen; HBsAb, Hepatitis B surface antibody; SD, standard deviation; IOR, interquartile range

As shown in Table 1, compared with male patients, female patients had higher baseline levels of γ-glutamyl transpeptidase (GGT) and ALT and shorter follow-up and treatment duration. However, there were no differences between the two groups with regard to baseline levels of total bilirubin, albumin, globulin, total bile acid, platelet count, AST, HBsAg and HBV DNA, baseline HbsAb status, age of treatment initiation, HBV genotype and treatment regimen.

### Dynamic change levels of HBsAg, hbeag and HBV DNA in children with chronic HBV infection between sexes

**Fig. 1:**
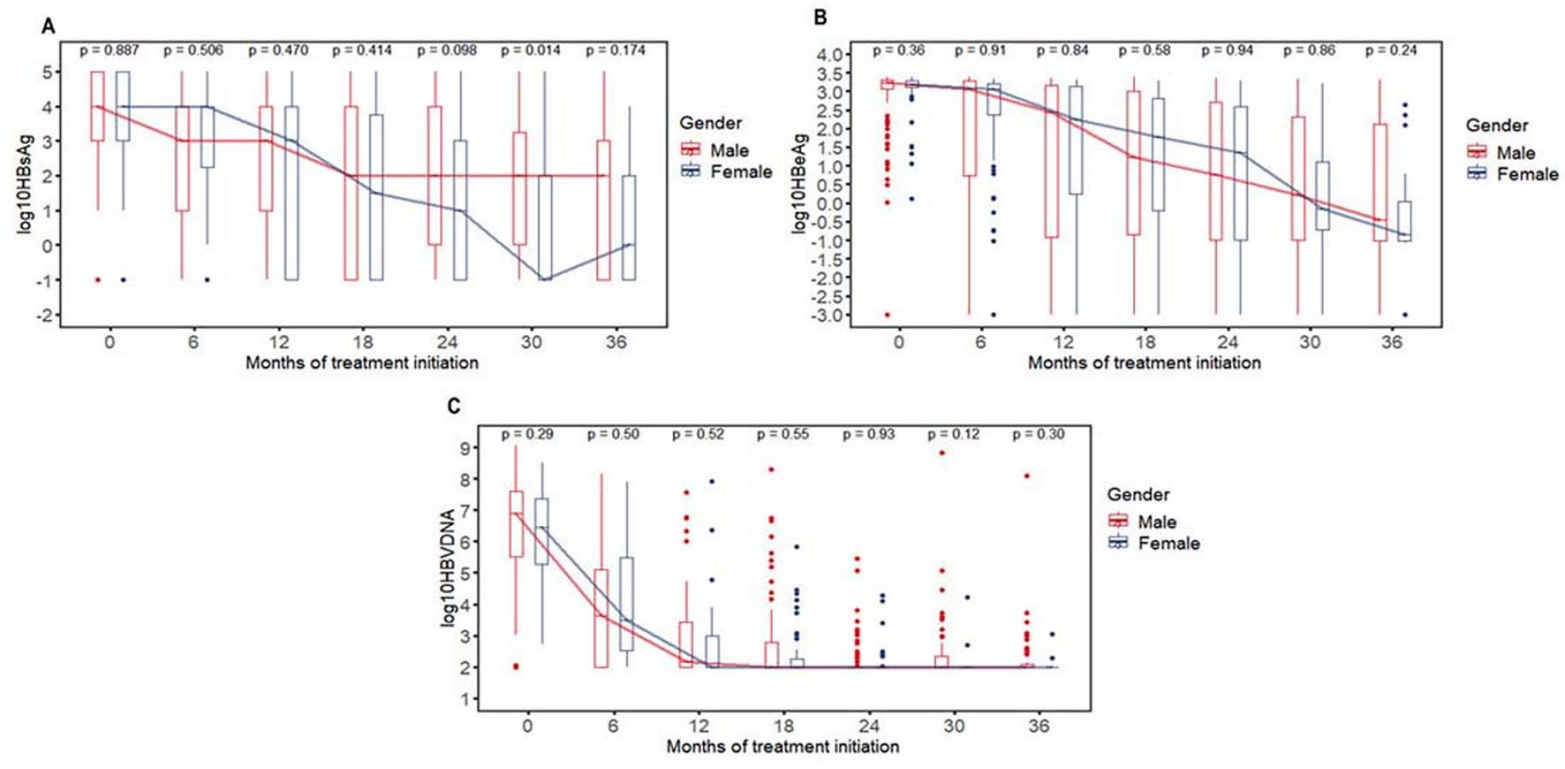
the levels of dynamic change of HBsAg (IU/ml) i, HBeAg (COI) and HBV DNA(IU/ml) in children with chronic HBV infection between different gender. Box plot shows 25%−75% observations around median (interquartile range, IQR)

Figure 1a shows that the decline in HBsAg levels was more pronounced in female patients than in male patients throughout the follow-up period, especially after 12 months. Median decreases in HBsAg levels in male patients were − 0.42/−1.07/−1.67/−1.96/−1.79/−2.28 log10 IU/mL at months 6, 12, 18, 24, 30 and 36 (*P* < 0.001 for differences from baseline) (Table S1). Median decreases in female patients were − 0.41/−1.62/−2.00/−2.91/−4.40/−3.11 log10 IU/mL (*P* < 0.001 for differences from baseline). However, no significant difference was observed in the decline of HBeAg and HBV DNA levels between male and female patients (see Table S1, Fig. 1b and c for details).

### Incidence of different clinical outcomes among children with chronic HBV infection by sex

During the entire follow-up period, 115 participants (48.73%) achieved HBsAg loss, 144 participants (61.02%) experienced HBeAg clearance, and 210 participants (89.36%) achieved HBV DNA undetectability. Notably, a higher cumulative incidence of HBsAg loss was observed in female patients compared to male patients (58.02% vs. 43.87%, *P* = 0.001, log-rank test). However, no significant difference was noted between male and female patients in terms of HBeAg clearance and HBV DNA undetectability (*P* > 0.05, log-rank test) (Table 2).

**Table 2:** Clinical outcomes in children with chronic HBV infection according to sex

Treatment responses	Total (*n* = 236)	Sex		*P* value
		Male	Female	
HBsAg loss, No (%)	115(48.73)	68(43.87)	47(58.02)	**0.001**
Median survival time of HBsAg loss(95%*CI*), mo	34.66(26.38,42.93)	42.09(23.79,60.40)	26.06(19.92,32.21)	
HBeAg clearance	144(61.02)	98(63.23)	46(56.79)	0.635
Median survival time of HBeAg clearance(95%*CI*), mo	26.50(21.56,31.44)	26.16(19.75,32.57)	24.31(16.51,32.11)	
HBV DNA undetectability, No (%)	210(89.36)	139(90.26)	71(87.65)	0.172
Median survival time of HBV DNA undetectability(95%*CI*), mo	12.16(10.97,13.35)	11.84(10.48,13.21)	12.47(10.72,14.22)	

The bold values indicate the better results than other filtering methods

HBsAg, Hepatitis B surface antigen; HBsAb, Hepatitis B surface antibody; *CI*: confidence interval

### The association of sex with different clinical outcomes in children with chronic HBV infection

**Fig. 2:**
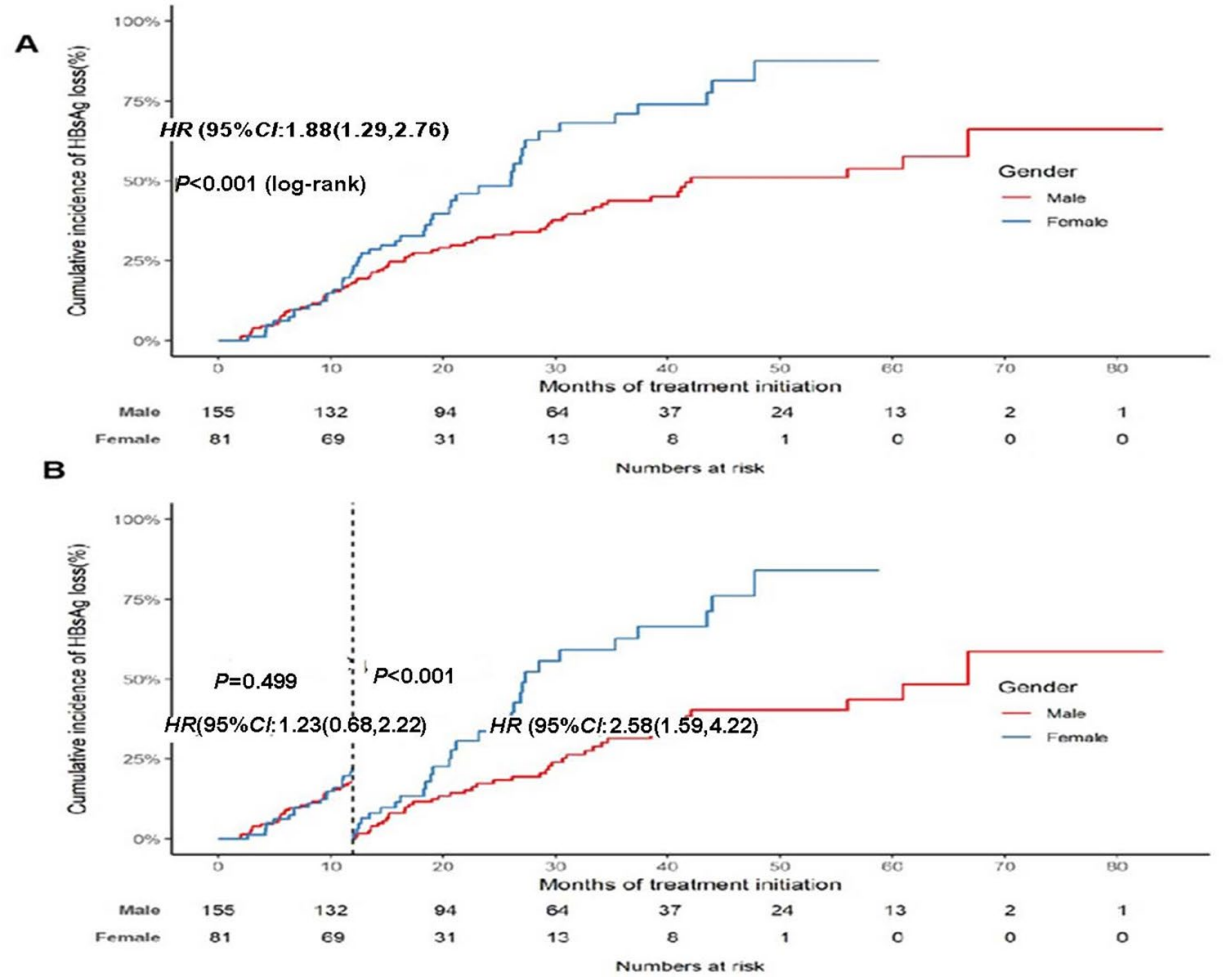
The K-M curve of HBsAg loss according to gender among children with chronic HBV infection. (**A**) Cumulative Incidence of HBsAg loss among children with chronic HBV infection. (**B**) Landmark analysis discriminating between events occurring before and after 1 year of treatment initiation

Multivariable analyses were conducted to identified the association between sex and treatment responses. Covariates included in multivariable analyses were determined based on the established association with treatment response in peer-reviewed literature, such as age, total bilirubin, globulin, albumin, total bile acid, GGT, ALT, AST, HBsAg, HBV DNA, and platelet count, baseline HBsAb status, and therapeutic regimen [9–11]. As shown in Table 3, it was observed that female patients had a higher probability of HBsAg loss compared to male patients throughout the follow-up period (adjusted HR (95%CI): 2.03(1.35–3.06)). However, there was no significant difference between the two groups in terms of HBeAg clearance and HBV DNA undetectability. Furthermore, given the intersection of the K-M curves for HBsAg loss between different sexes, it was suggested that the impact of sex on treatment response varied across different follow-up periods (Fig. 2). We further performed a landmark analysis on the clinical outcomes before and after the 1-year landmark points. The results showed no statistical difference in the incidence of HBsAg loss within the first year between male and female patients(*P* > 0.05). However, beyond the first year, female patients had a higher probability of HBsAg loss (adjusted HR: 2.18,95%CI: 1.29–3.69) (see Table 3; Fig. 2 for more details).

**Table 3:** The association of sex with different clinical outcomes in children with chronic HBV infection

Treatment responses	Male	Female	Unadjusted HR(95%CI)	*P* value	Adjusted HR(95%CI)^*^	*P* value
Whole follow-ups						
HBsAg loss	68(43.87)	47(58.02)	**1.88(1.29**,**2.76)**	**0.001**	**2.03(1.35**,**3.06)**	**< 0.001**
HBeAg clearance	98(63.23)	46(56.79)	1.09(0.76,1.56)	0.636	1.31(0.89,1.92)	0.175
HBV DNA undetecability	139(90.26)	71(87.65)	1.23(0.91,1.64)	0.173	1.28(0.94,1.74)	0.115
Landmark analysis (1 year)						
0–1 year						
HBsAg loss	28(18.06)	18(22.22)	1.23(0.68,2.22)	0.499	1.47(0.78,2.79)	0.233
> 1 year						
HBsAg loss	40(31.50)	29(46.77)	**2.58(1.58**,**4.22)**	**< 0.001**	**2.18(1.29**,**3.69)**	**0.004**

The bold values indicate the better results than other filtering methods

HBsAg, Hepatitis B surface antigen; HBeAg, Hepatitis B e antigen; HBsAb, Hepatitis B surface antibody; *HR*: Hazard ratio; *CI*: confidence interval

^*^adjustments for various factors, including age of treatment initiation, baseline levels of total bilirubin, globulin, albumin, total bile acid, γ-glutamyltranspeptidase, ALT, AST, HBsAg, HBV DNA, and platelet count, baseline HBsAb status, and therapeutic regimen

### Subgroups analysis stratifying by baseline ALT levels

Patients were categorized into two subgroups: ALT < 40 U/L and ALT ≥ 40 U/L. In the ALT < 40 U/L subgroup, females continued to exhibit significantly superior HBsAg clearance rates compared to males (HR [95% CI]: 3.62 [1.59–8.25]; *p* = 0.002). In the ALT ≥ 40 U/L subgroup, a trend favouring females was observed, though statistical significance was not reached (HR [95% CI]: 1.74 [1.00–3.04.00.04]; *p* = 0.052). Additionally, a formal interaction term (sex × baseline ALT stratum) was non-significant (P_interaction_=0.659), suggesting that the relationship between sex and treatment response is not statistically modified by baseline ALT levels (Table S2).

## Discussion

In this study, we investigated the association between sex and long-term treatment response in children with chronic HBV infection. In addition, the landmark method was used to further investigate differences in treatment response at different follow-up time points. Our results showed a significant correlation between sex and treatment response. Specifically, female patients were more likely to achieve HBsAg loss. However, this sex difference only became apparent after one year of follow-up. This suggests that sex may be an independent predictor of treatment responses after one- year of follow-up.

Previous studies have shown that achieving HBsAg loss, and HBeAg clearance in adults with chronic HBV infection are very difficult, especially HBsAg loss [17]. However, high treatment response rates have often been reported in paediatric patients. A retrospective real-world study of children with CHB who received 72 weeks of IFN-α therapy showed a high cumulative rate of HBsAg loss (46.95%) at 13 years of follow-up [18]. In addition, another study by Li et al. showed that of the 32 children with CHB who received single or combination therapy with interferon-α or a nucleoside analogue (NA), 18 (56.25%) achieved HBsAg loss during 36 months of follow-up [19]. Similarly, our study had also found that 48.73% of children with chronic HBV infection experienced HBsAg loss within a median follow-up of 27 months. These results suggest that antiviral treatment tends to lead to better outcomes in children with chronic HBV infection, making it particularly important to intensify treatment of children with chronic HBV infection to reduce the risk of developing cirrhosis and liver cancer and to ensure their long-term health.

Most importantly, our study showed that female patients were more likely to achieve HBsAg loss, which is consistent with the findings of previous studies by Mao and Jonas et al. [12, 13]. This observation may be attributed to sex differences in the immune response. In general, females exhibit stronger innate and adaptive immune responses compared to males, leading to a more rapid clearance of pathogens [20]. In addition, our study found that female patients had higher baseline levels of ALT, an indicator of the intensity of the immune response, which further supports this finding. Moreover, female patients exhibited better medication adherence to other infectious diseases [21, 22], which may contribute to the effectiveness and sustainability of treatment. This suggests that female individuals may have a higher preventive benefit against liver cancer and cirrhosis due to their better response to treatment and viral clearance.

However, it is noteworthy that the sex difference observed after one year of antiviral therapy was more pronounced. This may be due to the fact that the androgen receptor regulates epigenetic and transcriptional differentiation programmes, inhibiting the activity and function of CD8 + T cells in male patients, further reducing the therapeutic effect of interferon [23]. This finding has important implications for clinical practice, highlighting the need for long-term monitoring and evaluation of treatment outcomes, particularly when considering sex differences. For example, male patients who have a poor response to treatment within the first year may require closer monitoring or adjustment of treatment regimen. However, more research is needed to validate these findings and to better understand the mechanisms underlying sex differences in treatment response.

Our study has several limitations that should be acknowledged. First, this study was conducted as a retrospective cohort study at a single tertiary medical centre, which may introduce selection bias and limit the generalizability of our findings. Second, the data collected from existing medical records may have limitations in terms of capturing comprehensive information on important confounding factors such as genotype and medication adherence behaviours. Therefore, additional well-designed studies involving larger and more diverse populations are needed to further investigate and validate sex differences in treatment response among chronic HBV infection patients, taking into account various confounding factors. Last, liver fibrosis staging was not systematically assessed due to the ethical and practical constraints of performing invasive biopsies in children, which merits consideration as fibrosis severity may influence therapeutic outcomes.

## Conclusion

In conclusion, our study shows significant differences in the responses to antiviral treatment between male and female chronic HBV-infected children who received monotherapy or combination therapy with interferon/peginterferon and entecavir. Our finding suggests that female patients are more likely to achieve HBsAg loss, especially after one year of follow-up. These findings underscore the importance of longer-term monitoring and evaluation of treatment outcomes, particularly in male patients who may experience slower or less significant progress.

## Supplementary Information

Supplementary Material 1

## Data Availability

Because the data presented is part of several ongoing projects, availability of data will be made available by request.
