# Supplementary material for: Sex differences in the clinical outcomes of chronic hepatitis B infection among paediatric patients
Source: Biol Sex Differ. 2026 Mar 25;17:95. doi: 10.1186/s13293-026-00826-8 (PMC13141448; doi:10.1186/s13293-026-00826-8)
Supplement: Supplementary file 1 — Supplementary Material 1 [file 13293_2026_826_MOESM1_ESM.docx]

| Table S1 The dynamic change levels of HBsAg, HBeAg and HBV DNA in children with HBV infection between male and female | | | | | | | | | | | | |
| --- | --- | --- | --- | --- | --- | --- | --- | --- | --- | --- | --- | --- |
|  | T_6moth_-T_baseline_ | | T_12moth_-T_baseline_ | | T_18moth_-T_baseline_ | | T_24moth_-T_baseline_ | | T_30moth_-T_baseline_ | | T_36moth_-T_baseline_ | |
|  | Median Declines | *P* value | Median Declines | *P* value | Median Declines | *P* value | Median Declines | *P* value | Median Declines | *P* value | Median Declines | *P* value |
| HBsAg(log10 IU/ml)) | | |  |  |  |  |  |  |  |  |  |  |
| Male | -0.42 | 0.652 | -1.07 | 0.120 | -1.67 | 0.120 | -1.96 | 0.092 | -1.79 | 0.013 | -2.28 | 0.087 |
| female | -0.41 |  | -1.62 |  | -2.00 |  | -2.91 |  | -4.40 |  | -3.11 |  |
| HBeAg(log10 coi) | | |  |  |  |  |  |  |  |  |  |  |
| Male | -0.12 | 0.215 | -0.46 | 0.652 | -1.77 | 0.823 | -2.26 | 0.611 | -2.90 | 0.692 | -2.91 | 0.149 |
| female | -0.12 |  | -0.52 |  | -1.43 |  | -1.59 |  | -3.45 |  | -4.10 |  |
| HBV DNA(log10 IU/ml) | | |  |  |  |  |  |  |  |  |  |  |
| Male | -2.66 | 0.700 | -3.88 | 0.655 | -4.46 | 0.933 | -4.66 | 0.839 | -4.74 | 0.786 | -4.87 | 0.704 |
| female | -2.46 |  | -4.00 |  | -4.35 |  | -4.50 |  | -5.04 |  | -4.70 |  |

Table S2. Associations between sex and HBsAg loss in children with chronic HBV infection stratified by ALT.

| Characteristics | Males | Females | aOR (95% CI) | *P* | P_interaction_ |
| --- | --- | --- | --- | --- | --- |
| Overall | 155 | 81 | 2.03(1.35,3.06) | <0.001 |  |
| ALT |  |  |  |  |  |
| <40 | 18/54(33.3) | 20/36(55.6) | 3.62 (1.59-8.25) | 0.002 | 0.659 |
| ≥40 | 50/101(49.5) | 27/45(60.0) | 1.74 (1.00-3.04) | 0.052 |  |

aOR, adjusted for age of treatment initiation, baseline levels of total bilirubin, globulin, albumin, total bile acid, γ-glutamyltranspeptidase, AST, HBsAg, HBV DNA, and platelet count, baseline HBsAb status, and therapeutic regimen.
